# Risk factors for sexually transmitted infections among men who have sex with men

**DOI:** 10.4102/phcfm.v15i1.4080

**Published:** 2023-10-17

**Authors:** Matshidiso A. Malefo, Olalekan Ayo-Yusuf, Mathildah M. Mokgatle

**Affiliations:** 1 School of Health Care Sciences, Faculty of Health Sciences, Sefako Makgatho Health Sciences University, Pretoria, South Africa; 2 School of Health Systems and Public Health, Faculty of Health Sciences, University of Pretoria, Pretoria, South Africa

**Keywords:** men who have sex with men, asymptomatic, sexually transmitted infections, risk factors, sexual behaviour, alcohol use, HIV

## Abstract

**Background:**

Sexually transmitted infections (STIs) are a global public health concern and sub-Saharan Africa, has limited data on STIs in the men who have sex with men (MSM) population. Syndromic management has controversies for treating asymptomatic STIs (ASTIs).

**Aim:**

The aim of this study was to describe the risk factors for STIs among MSM.

**Setting:**

This study was conducted in Tshwane North, Gauteng Province in South Africa.

**Methods:**

A cross-sectional quantitative design was employed using structured questionnaires, rapid plasma reagent test, from December 2021 to May 2022. Bivariate and multivariate analyses were used for statistical analysis.

**Results:**

A total of 200 MSM with the mean age of 27.6, standard deviations: 6.8 participated, and STIs prevalence was 66%, with 37% concurrent infections. *Ureaplasma urealyticum* was (24%), Mycoplasma *hominis* (23%), *Chlamydia trachomatis* (20%), *Treponema pallidum* (20%) and *Neisseria gonorrhoeae* (9%). The risk factors for acquisition of STI include having a new partner in the last month (OR = 1.68; CI: 0.98–3.13).

**Conclusion:**

The prevalence of ASTIs is high. Serial and multiple sexual partners are the risk factors.

**Contribution:**

This study contributes to the body of knowledge of the burden of STIs among high-risk population.

## Introduction

Sexually transmitted infections (STIs) are a major public health concern. They may increase the risk of contracting human immunodeficiency virus (HIV) through receptive and insertive anal intercourse.^1,2^ In South Africa, the prevalence of reported asymptomatic STIs (ASTIs) varies wildly, ranging from 19% to 90%.^3,4,5^ Among the other drivers of STIs among men who have sex with men (MSM) such as risky behaviours, undetected or ASTIs might be an important contributor to the high prevalence of STIs.^3^ Infection at anorectal sites among MSM might be symptomatic or asymptomatic. Asymptomatic disease is more likely to be inadequately diagnosed and treated.^6,7^ The Sibanye Health Project reported that 91%, 95% and 97% of diagnosed rectal *Neisseria gonorrhoeae* (NG)and/or *Chlamydia trachomatis* (CT),urethral NG and CT and syphilis infections, respectively, were clinically asymptomatic among South African MSM.^8^ Despite most STIs such as gonorrhoea and chlamydia being curable, a delayed diagnosis is associated with a worse prognosis.^9^

The data collected from Europe, the United States and China indicated that MSM have a high burden of HIV and other STIs.^10^ For example, in England in 2019, there were 77 371 new STI diagnoses in MSM.^11^ Of the new cases recorded among MSM in 2019, NG (*n* = 33 853; 44%) and CT (*n* = 23 187; 30%) were the most common. In the same year, there were 5875 (8%) new cases of syphilis in MSM.^11^ In comparison, there were 97 450 new STI diagnoses among non-MSM males.^11^ Chlamydia was more common among non-MSM males (*n* = 46 192; 47.4%) followed by NG (*n* = 15 253; 15.7%). There were 1016 (1%) new syphilis infections. The number of new gonorrhoea cases diagnosed in 2019 (*n* = 70.936) was the largest annual number reported since records began in 1918, and 33 853 (47.7%) of those cases were reported in MSM.^11^

Social stigma and punitive civil environments may lead to delays in seeking HIV and STI screening and consequently later initiation of treatment.^12^ Serosorting, HIV pre-exposure prophylaxis, an increase in unprotected sexual intercourses between sexual partners and recreational drug use may all have an impact on STI transmission, primarily in asymptomatic carriers.^13,14,15,16,17^ Spinner et al. reported high numbers of asymptomatic syphilis, hepatitis C, CT and gonorrhoea in MSM with a shorter duration of HIV infection and having more than one sexual partner within the last six months.^13^ The study conducted in South Africa among MSM showed that asymptomatic infection was associated with transgender identity, having five male sex partners in the last year, and transactional sex, but not with HIV infection.^3^

The guidelines for STI treatment in South Africa employ the syndromic management approach.^18^ Syndromic management relies on individuals recognising the signs and symptoms of STIs, seeking healthcare and reporting the symptoms to healthcare professionals. Although screening for ASTIs is recommended as part of HIV prevention efforts, optimal screening strategies among HIV-infected MSM are unknown. Rectal STI screening is less frequently performed in the health facilities than urethral screening among MSM. The South African STI guidelines do not include guidance on how to manage anorectal discharge as a syndrome.^3^ The HIV prevention and STI interventions are primarily aimed at the heterosexual population, but MSM require special consideration. Pharyngeal and rectal infections in sexually active MSM could remain undetected and thus transmissible if screening is not routinely offered. Data are needed in order to understand the risk factors for ASTIs in MSM for the implementation of targeted STI screening strategies.

Hence, the study sought to assess the risk factors for STIs among MSM.

## Research methods and design

### Study design

The study conducted cross-sectional baseline data which were nested in the longitudinal study from December 2021 to May 2022 and enrolled asymptomatic MSM.

### Study setting and population

This study was carried out in Ga-Rankuwa, Tshwane North, Gauteng province in South Africa. Participants in the study were recruited from December 2021 to May 2022. Men were eligible if they were 18 years of age or older and had anal intercourse with one or more men in the previous year. Outreaches in the streets, dance clubs, bars, sex clubs, health clubs and adult video stores were among the recruitment strategies used. Participants were also recruited through community forums and snowballing.

Studies conducted among the MSM community have reported the lack of robust MSM population size estimates,^19^ which is suggestive of a respondent-driven sampling (RDS) technique. Respondent-driven sampling is a snowball sampling technique that will be suitable for this study. This is a method which has been used in MSM research in other countries.^20,21^ Respondent-driven sampling is a referral-based system in which participants refer friends and acquaintances who are also member of target population to participate in the study.

Previous studies and trial conducted among MSM in Medunsa Clinical Research Unit (MeCRU), which is a setting for this research, enrolled approximately 200 MSM participants and our study plans to achieve 377, which is a sample size calculated by the Raosoft sample size calculator using a value of unknown population size.^22^ The sample is estimated with a margin error of 5% and a confidence interval of 95%. The sample size for the proposed study is based on the calculation of precision in the proportion of MSM who completed the 12-month scheduled visits. The authors estimate that a successful attendance must demonstrate greater than 90% completion of the entire 12-month scheduled.

### Data collection and measures

Following informed consent, a self-administered questionnaire was used to collect demographic and behavioural data. The participants reported their age, gender and sexual orientation, as well as their highest educational attainment and employment status. Sexual risk factors including receptive condomless anal intercourse, the number of sex partners, transactional sex and alcohol use were among the relevant behavioural variables. The questionnaire was administered in the participants’ preferred languages, English or Setswana.

All study participants were offered STI screening. A clinician used direct swabbing to obtain samples for testing from rectal, oral and genital swabs. Participants who received an STI diagnosis were referred to a local clinic based on their laboratory results. The participants were tested for the following: NG, CT, *Mycoplasma genitalium* (MG) and *Mycoplasma hominis* (MH), *Trichomonas vaginalis* (TV), *Ureaplasma urealyticum* (UU) and *Ureaplasma parvum* (UP), *Treponema pallidum* (TPHA) and syphilis.

### Data analysis

The data were analysed using Stata 17.0 (Stata Corporation, College Station, TX, USA). The data were summarised using descriptive statistics (proportions and means with standard deviations [s.d.]). Nonparametric tests such as Wilcoxon signed-rank test and stepwise forward logistic regression were used to determine the risk factors. Logistic regression models were set at 95% confidence intervals. Statistical significance was determined at *p* ≤ 0.05. The dependent variable was positive STI diagnosis and the independent variables were age, source of income, known HIV-positive sex partner status, type of relationship, sexual orientation, number of sexual partners in the last month, and prevention method during sex, sex under influence, transaction sex, tested for STI last 3 months, and tested for HIV last 3 months.

### Ethical consideration

Ethical clearance and approval to conduct this study was obtained from the Sefako Makgatho Health Sciences University Research Ethics Committee (No. SMUREC/H/168/2019:PG). The study was conducted in accordance with the Helsinki Declaration. All participants provided written informed consent. Participants’ specimens were labelled with a unique identity number (range from 1 to 200) to enable linkage to the risky sexual behaviour survey.

## Results

### Sociodemographic characteristics of the men who have sex with men

Table 1 summarises the baseline characteristics of the participants as well as the range of their sexual activities and risk behaviours. The study included 200 MSM as participants. Their average age was 27.6 years (s.d. 6.9). Most of the participants were black (99%), had no job (65%) and had finished high school (84%).

**TABLE 1:** Baseline characteristics and sexual behaviours.

Variables	Frequency	Percentage
**Baseline characteristics**		
**Age group**		
15–25 years	85	42.71
26 and above	114	57.29
**Highest level of schooling**		
High school or higher	183	91.50
Primary or none	17	42.71
**Race**		
Black people	198	99.0
Mixed race people	2	1.0
**Employment status**		
Employed	70	35.0
Unemployed	130	65.0
**Type of relationship**		
Stable	85	42.5
Casual	115	57.5
**Sexual behaviour characteristics and other risk factors for STIs**		
**Sexual orientation**		
Bisexual	57	28.50
Gay	143	71.50
**Sexual partners in the last month**		
One	90	45.0
Two or more	110	55.0
**Prevention method during sex**		
Condoms	71	35.5
Lubricant	36	18.0
**Sex under the influence of alcohol, last 3 months**		
Yes	131	65.5
No	69	34.5
**Sex in exchange for money, last 3 months**		
Yes	68	34.0
No	132	66.0
**Sexual practice**		
**Anal receptive**		
Yes	84	42.0
No	116	58.0
**Anal insertive**		
Yes	141	70.5
No	58	29.0
Missing	1	0.5
**Vaginal sex**		
Yes	34	17.0
No	166	83.0
**Rimming bottom**		
Yes	39	19.5
No	161	80.5
**Rimming top**		
Yes	39	19.5
No	161	80.5
**Oral receptive**		
Yes	72	36.0
No	128	64.0
**Oral insertive**		
Yes	61	30.5
No	139	69.5

STIs, sexually transmitted infections.

### Sexual behaviour characteristics of the participants

Most participants (57.5%) had casual partners, with 28.5% having three or more sex partners in the last month. According to their sexual orientation, 63.5% were gay, with bisexuals coming in second (25%). In their lifetime, 66.5% of the participants reported having sex with a male partner, while 31.1% reported having sex with both male and female partners. A total of 37% of the participants used both condoms and lubricants during their last sexual relationship, while 34% used male condoms only. In the past 3 months, 65.5% of the individuals had sex while under the influence of alcohol, while 34% had transactional sex. Most participants (95.5%) had anal receptive or insertive sex; 83% had vaginal sex; 80% had had rimming bottom or top; 64.0% had oral receptive sex; and 69.5% had oral insertive sex.

### Sexually transmitted infection prevalence, differences in proportions and associated factors

*Ureaplasma urealyticum* was the most common STI (24%, *n* = 48), followed by *M. hominis* (23%, *n* = 46), CT (20%, *n* = 40), TPHA (20%, *n* = 40) and NG (36%, *n* = 18).

**FIGURE 1:**
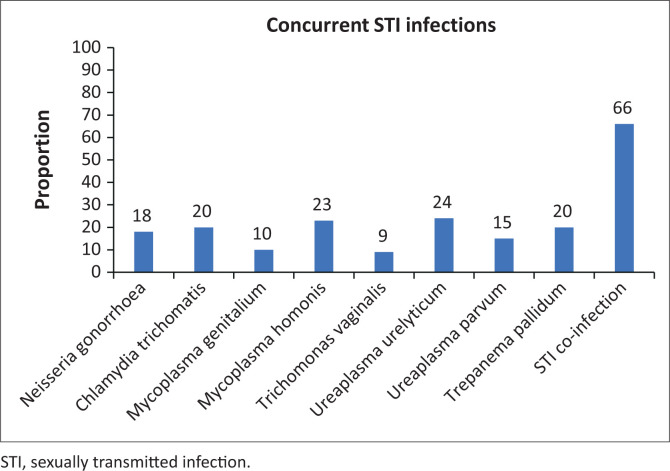
Prevalence of sexually transmitted infections in men who have sex with men sample.

Two-thirds of the participants (66%, *n* = 132) were infected with one or more types of STI organisms and 34% (*n* = 68) tested negative for STIs (Figure 1). As indicated in Table 2, 29% (*n* = 58) of the participants tested positive for only one STI, 19% (*n* = 38) for two STIs, 13% (*n* = 26) for three STIs, 3% (*n* = 6) for four STIs and 2% (*n* = 4) for five STIs.

**TABLE 2:** Number of sexually transmitted infection micro-organisms per specimen among men who have sex with men.

Number of STIs per participant	Frequency	Percentage
0 or negative test	68	34
1	58	29
2	38	19
3	26	13
4	6	3
5	4	2

**Total**	**132**	**66**

STIs, sexually transmitted infections.

### Factors associated with sexually transmitted infection acquisition

A Wilcoxon signed-rank test showed that age group, transactional sex and being tested for HIV were statistically significant with the acquisition of STIs in MSM (*p* = 0.0000). Additionally, the number of sexual partners in the last month, partner’s HIV status and being tested for STIs in the last three months were statistically significant with the acquisition of STIs (*p* < 0.05) (Table 3). The odds ratio (OR = 1.68, CI = 0.90, 3.14) for the number of sexual partners in the last month indicated that a person who had had more than one sexual partner was 1.6 times more likely to have acquired an ASTI. Similarly, men who did not use a condom as a prevention method were 1.26 times more likely to have acquired an ASTI than men who used a condom for prevention (OR = 1.26. CI = 0.66, 2.41) (Table 4).

**TABLE 3:** Wilcoxon signed-rank test of men who have sex with men diagnosed with sexually transmitted infections.

Variable	Diagnosed with STI				*p*-value
	Yes		No		
	*n*	%	*n*	%	
**Age group**					
15–25 years	55	41.98	30	44.12	0.0000
26 and above	76	58.02	38	55.88	-
**Education**					
Primary	11	8.33	6	8.82	1.000
High school and above	121	91.67	62	91.18	-
**Sexual orientation**					
Bisexual	38	28.79	19	27.94	0.900
Gay	94	71.21	49	72.06	-
**Relationship type**					
Casual	73	55.30	42	61.76	0.111
Stable	59	44.70	26	38.24	-
**Gender had sex with**					
Male	90	68.18	43	63.24	0.528
Both	42	31.82	25	36.76	-
**Sexual partners in the last month**					
One	55	41.47	35	51.47	**0.025**
Two or more	77	58.33	33	48.52	-
**Partner’s HIV status**					
One	22	16.67	10	14.71	0.049
Two or more	17	12.88	10	14.71	-
None	93	70.45	48	70.59	-
**Prevention method**					
Condom use	44	33.33	27	39.71	0.284
Non-condom use	88	66.67	41	60.29	-
**Sex under the influence**					
Yes	84	63.64	41	60.29	1.0000
No	48	36.36	27	39.71	-
**Transactional sex**					
Yes	43	32.58	25	36.76	0.0000
No	89	67.42	43	63.24	-
**Tested for HIV**					
Yes	116	87.87	50	73.53	0.0000
No	16	12.12	18	26.47	-
**Tested for STIs last three months**					
Yes	19	14.39	17	25.0	0.0002
No	113	85.60	51	75.0	-

STI, sexually transmitted infection.

**TABLE 4:** Logistic regression risk factors for acquisition of sexually transmitted infections for men who have sex with men (*n* = 200).

Variables	STI acquisition – Any STI			
	UOR	95% CI	AOR	95% CI
**Age**			
15–25 years	0.92	0.51–1.66	0.93	0.50–1.72
26 and above	Ref.	-	Ref.	-
**Source of income**			
Employed	Ref.	-	-	-
Not working	0.76	0.41–1.41	0.80	0.41–1.55
**Knowledge of last partner’s HIV status**				
One	Ref.	-	Ref.	-
Two or more	0.77	0.26–2.28	0.71	0.23–2.17
None	0.88	0.39–2.01	0.96	0.41–2.25
**Type of relationship**			
Stable	Ref.	-	Ref.	-
Casual	0.77	0.42–1.39	0.72	0.39–1.35
**Sexual orientation**			
Bisexual	Ref.	-	Ref.	-
Gay	0.96	0.50–1.84	0.94	0.47–1.88
**Sexual partners in the last month**				
One	Ref.	-	Ref.	-
Two or more	1.48	0.82–2.667	1.68	0.90–3.14
**Prevention method during sex**				
Condoms	Ref.	-	Ref.	-
Non-condom use	1.32	0.72–2.41	1.26	0.66–2.4

Note: Ref. refers to the observed baseline value for the specified variable. The reference level is omitted because it would have “0” for each observation.

STI, sexually transmitted infection; HIV, human immunodeficiency virus.

## Discussion

The prevalence of ASTI among the MSM was found to be high, and most MSM were infected with multiple organisms including *U. urealyticum, M. hominis, C. trachomatis, T. pallidum, N. gonorrhoeae.* The 66% STI prevalence in this study is relatively low compared with a study conducted in another city in Gauteng province, South Africa,^3,5,19^ however, higher than in other studies conducted in Kenya, Thailand and Tanzania. Rebe and colleagues^3^ reported that 24% of MSM in South Africa screened positive for NG and/or CT at any site. The results of the study conducted in Thailand found that gonorrhoeal prevalence for male genital site, anal and oropharyngeal, was 34.73%, while 5.9% tested positive for gonococcal infection in all anatomic sites.^23^ Similarly, one study of MSM from Kenya found that 26% tested positive for NG, CT or both.^7,24^ A study of 172 MSM in two Tanzanian cities found NG, CT and syphilis rates of 21%.^25^ This suggests that the burden of STIs among the MSM in South Africa, specifically in Gauteng province, is higher compared with other countries in the region.

The prevalence reported in the current study population is also higher than in the United States, where the prevalence rates of asymptomatic infection have been reported to be 38% and 56% concurrent infections among 326 MSM attending an STI clinic.^26^ This rate is significantly higher than the 10% reported in another study of MSM in South Africa.^8^ Ye and colleagues^27^ found that 80% of 177 MSM enrolled in their study had an STI burden of whom 30% had a sexually transmitted co-infection. The study conducted in Melbourne, Australia, among MSM attending a sexual health clinic found that MG was detected in 7% of asymptomatic participants at the rectum, 2.7% at the urethra and only 0.2% at both the sites.^28^

The results of this study indicated that 29% of participants were diagnosed with at least one STI. This relatively high prevalence is possibly due to the fact that up to 90% of STIs do not cause any symptoms,^29^ suggesting that a testing method based on symptoms would not be able to find STIs correctly or stop the spread of the disease through prompt treatment. The inability to diagnose ASTIs early leads to undertreatment, poor STI control and possibly HIV transmission in this high-risk population, and it presents as a missed opportunity to treat and control the spread.^3^ Previous research has shown that testing for a single STI is accompanied by a high proportion of missed diagnoses for other STIs and that the population of MSM continue to be under-diagnosed for STIs.^30,31^

The increased STI burden is unlikely to be because of an undiagnosed reservoir of STIs. High-risk sexual behaviour remains a significant barrier to STI control among MSM. Our findings show that MSM engage in risky sexual behaviours. Fifty-five percent of the enrolled participants had more than one sexual partner in the previous month; 1 in 10 had sex without any protection; roughly two-thirds had had sex under the influence of alcohol; and 34% had sex for money in the previous 3 months. These findings highlight the importance of MSM-focused HIV and STI prevention interventions, which are multifaceted and include increased testing, prompt treatment and the ongoing promotion of safer sexual behaviour among MSM.^32^

In this study, the most prominent risk behaviour for STI acquisition was having more than one sexual partner in the past month. This finding implies that having serial partners, multiple partners and being in an unstable sexual relationship predisposed the MSM to infections and could compromise protective behaviours for prevention of STIs. A follow-up study of MSM discovered that self-reported behavioural information such as unsafe sex practices, having partners who practise unsafe sex and alcohol use were not predictive of the incidence of STIs, and even without confirmed association, the MSM in this study displayed similar behaviours.^6^ There might be other risk factors for STI acquisition in this understudied population in South Africa that we probably have not included such as the history of illicit drug use and the CD4 count related to the HIV status, both of which have been associated with a higher incidence of ASTIs. Furthermore, Reig et al.^6^ reported that a lack of association of positive STI diagnosis and significant predictors at bivariate analysis would be related to the tendency of the MSM to underreport these risky sexual behaviours in a clinical setting based on the sensitivity and social desirability. Appropriate attention should be given to the MSM population, such as focusing on education regarding prevention measures, especially persistent condom use.^27^

Further research into the relationship between sexual and behavioural risks and psychosocial risk factors is required, using a longitudinal study design with a large sample size comparing risk factors among MSM who develop symptomatic STIs to those with ASTIs.

### Limitations

The study has some limitations. To begin with, the cross-sectional analyses included STI diagnoses at a single point in time only. Because cross-sectional studies do not identify newly acquired infections, longitudinal studies of the incidence of ASTIs are important. Previous research has shown that NG and CT can live in asymptomatic people for months, making them persistent reservoirs of infection.^6^ Another limitation of our study was the small sample size, which limited the statistical power to investigate further stratified associations during the analysis of the study data. Furthermore, the participants in the study may not be representative of all MSM in South Africa. Despite the limitations of this study, it represents one of the few such published studies from resource-poor settings in a population of MSM.

## Conclusion

Asymptomatic STIs were common and would have gone undetected if a syndromic management approach had been used. The high rate of STI co-infections is especially concerning, indicating that people do not perceive themselves at risk for another STI. As part of the established HIV prevention efforts, the regular testing and treatment of asymptomatic MSM is a crucial element of effective control. It is important to obtain accurate STI surveillance data to develop prevention programmes and measures to evaluate the effect of behavioural interventions among the MSM population.
