# Corrigendum: Risk factors for sexually transmitted infections among men who have sex with men

**DOI:** 10.4102/phcfm.v16i1.4660

**Published:** 2024-06-28

**Authors:** Matshidiso A. Malefo, Olalekan Ayo-Yusuf, Mathildah M. Mokgatle

**Affiliations:** 1 School of Health Care Sciences, Faculty of Health Sciences, Sefako Makgatho Health Sciences University, Pretoria, South Africa; 2 School of Health Systems and Public Health, Faculty of Health Sciences, University of Pretoria, Pretoria, South Africa

In the published article, Malefo MA, Ayo-Yusuf O, Mokgatle MM. Risk factors for sexually transmitted infections among men who have sex with men. Afr J Prm Health Care Fam Med 2023;15(1):a4080. https://doi.org/10.4102/phcfm.v15i1.4080, there were statistical data errors in the published article. *Neisseria gonorrhoea* was incorrectly set as ‘(9%)’ in the abstract, as well as *Neisseria gonorrhoeae* (NG) was incorrectly set as (36%; *n* = 18).

The correction has now been made to correct the results section of the article’s abstract, on page 1 of 7. The original paragraph read:

**Results:** A total of 200 MSM with the mean age of 27.6, standard deviations: 6.8 participated, and STIs prevalence was 66%, with 37% concurrent infections. *Ureaplasma urealyticum* was (24%), *Mycoplasma hominis* (23%), *Chlamydia trachomatis* (20%), *Treponema pallidum* (20%) and *Neisseria gonorrhoeae* (9%). The risk factors for acquisition of STI include having a new partner in the last month (OR = 1.68; CI: 0.98–3.13).

The revised and updated paragraph should read:

**Results:** A total of 200 MSM with the mean age of 27.6, standard deviations: 6.8 participated, and STIs prevalence was 66%, with 37% concurrent infections. *Ureaplasma urealyticum* was (24%), *Mycoplasma hominis* (23%), *Chlamydia trachomatis* (20%), *Treponema pallidum* (20%) and *Neisseria gonorrhoeae* (18%). The risk factors for acquisition of STI include having a new partner in the last month (OR = 1.68; CI: 0.98–3.13).

There was another error in paragraph number 4, on page 3 of 7, under the sub-heading, ‘Sexually transmitted infection prevalence, differences in proportions and associated factors’ section. The original paragraph read:

*Ureaplasma urealyticum* was the most common STI (24%, *n* = 48), followed by *M. hominis* (23%, *n* = 46), CT (20%, *n* = 40), TPHA (20%, *n* = 40) and NG (36%, *n* = 18).

The revised and updated paragraph should read:

*Ureaplasma urealyticum* was the most common STI (24%, *n* = 48), followed by *M. hominis* (23%, *n* = 46), CT (20%, *n* = 40), TPHA (20%, *n* = 40) and NG (18%; *n* = 36)

The authors apologise for this error. The correction does not change the study’s findings, its significance or overall interpretation of its results or the scientific conclusions of the article in any way.

